# Seed Transmission of Pathogens: Non-Canonical Immune Response in *Arabidopsis* Germinating Seeds Compared to Early Seedlings against the Necrotrophic Fungus *Alternaria brassicicola*

**DOI:** 10.3390/plants11131708

**Published:** 2022-06-28

**Authors:** Mailen Ortega-Cuadros, Tiago Lodi De Souza, Romain Berruyer, Sophie Aligon, Sandra Pelletier, Jean-Pierre Renou, Tatiana Arias, Claire Campion, Thomas Guillemette, Jérome Verdier, Philippe Grappin

**Affiliations:** 1 Faculty of Exact and Natural Sciences, Institute of Biology, University City Campus, University of Antioquia, Calle 67 N°53-108, Medellín 050010, Colombia; mailen.ortega@udea.edu.co; 2 Institut Agro, University Angers, INRAE, IRHS, SFR 4207 QuaSaV, F-49000 Angers, France; tiago.lodidesouza@pl.chambagri.fr (T.L.D.S.); romain.berruyer@univ-angers.fr (R.B.); sophie.aligon@agrocampus-ouest.fr (S.A.); sandra.pelletier@inrae.fr (S.P.); jean-pierre.renou@inrae.fr (J.-P.R.); claire.campion@univ-angers.fr (C.C.); thomas.guillemette@univ-angers.fr (T.G.); jerome.verdier@inrae.fr (J.V.); 3 Marie Selby Botanical Gardens, Downtown Sarasota Campus, 1534 Mound Street, Sarasota, FL 34236, USA; tarias@selby.org

**Keywords:** *Arabidopsis*, *Alternaria brassicicola*, seed-borne pathogen transmission, susceptible response

## Abstract

The transmission of seed-borne pathogens by the germinating seed is responsible for major crop diseases. The immune responses of the seed facing biotic invaders are poorly documented so far. The *Arabidopsis thaliana*/*Alternaria brassicicola* patho-system was used to describe at the transcription level the responses of germinating seeds and young seedling stages to infection by the necrotrophic fungus. RNA-seq analyses of healthy versus inoculated seeds at 3 days after sowing (DAS), stage of radicle emergence, and at 6 and 10 DAS, two stages of seedling establishment, identified thousands of differentially expressed genes by *Alternaria* infection. Response to hypoxia, ethylene and indole pathways were found to be induced by *Alternaria* in the germinating seeds. However, surprisingly, the defense responses, namely the salicylic acid (SA) pathway, the response to reactive oxygen species (ROS), the endoplasmic reticulum-associated protein degradation (ERAD) and programmed cell death, were found to be strongly induced only during the latter post-germination stages. We propose that this non-canonical immune response in early germinating seeds compared to early seedling establishment was potentially due to the seed-to-seedling transition phase. Phenotypic analyses of about 14 mutants altered in the main defense pathways illustrated these specific defense responses. The unexpected germination deficiency and insensitivity to *Alternaria* in the glucosinolate deficient mutants allow hypothesis of a trade-off between seed germination, necrosis induction and *Alternaria* transmission to the seedling. The imbalance of the SA and jasmonic acid (JA) pathways to the detriment of the JA also illustrated a non-canonical immune response at the first stages of the seedling.

## 1. Introduction

Because the seed is an important component of plant propagation, it is used as raw material for agriculture [1] and can also be the initial stage for pathogens transmission to the plant allowing pathogen dissemination and spreading [2,3,4]. Seed-borne pathogens can be both detrimental to seed vigor and a major cause of disease outbreaks [4], so controlling seed health quality and seed immune response is critical in the agricultural sector [5].

The mechanisms of plant resistance have been extensively studied for many years [6]. Overall, there is a co-adaptation between the plant immune system that confers the capacity to recognize and respond to a pathogen and the pathogen that in turn develops strategies to manipulate host immunity and cause diseases [6,7]. Two major innate immune systems based on the recognition of non-self components have been developed to overcome attacks, which lead to similar defense responses. One is based on the pattern recognition receptors (PRRs) at the cell surface of the plants. The PRRs confer an exceptional capacity to recognize pathogens through their microbial degradation products such as cell wall fragments or through their elicitors such as fungal chitin or bacterial flagellin, which are called, respectively, damage-associated molecular patterns (DAMPs) and pathogen/microbe-associated molecular patterns (P/MAMPs). This recognition triggers the host defense response, PAMP-triggered immunity (PTI), that is very effective in resistance against non-adapted (non-host) pathogens. The second occurs through host intracellular receptors, namely nucleotide binding (NB) leucine-rich repeat (LRR) receptor (NLR) proteins, which are able to detect pathogen effectors and induce effector-triggered immunity (ETI). The latter system is highly subjected to co-adaptation as the result of receptor and effector diversification. ETI is rather reactive against host-pathogens and often involves a localized cell death, called the hypersensitive response (HR). Immune responses activate physical barriers such as waxy cuticles, callose deposition in cell walls, and antimicrobial secondary metabolites, reactive oxygen species (ROS) burst, along with hormonal pathways (i.e., salicylic acid (SA), jasmonic acid (JA), ET signaling) and antimicrobial secondary metabolites [6,8,9,10]. Previously challenged plants could develop a long-lasting systemic acquired resistance (SAR) [11,12], which propagates a signal for resistance against a broad spectrum of pathogens in non-inoculated tissues of the infected plant [13]. SAR is known to be induced by SA and pipecolic (Pip) pathways through a long-distance signal [14]. The plant pathogen induces SAR through tissue necrosis either as a part of HR or as a symptom of a disease. In some cases, the pathogen virulence evolves and overcomes the plant defenses leading to disease symptoms and pathogen spread into the host [15]. This is the case when the pathogen effector proteins are able to suppress the MAMP-activated defenses so that the pathogen establishes effector-triggered susceptibility (ETS) by suppressing PTI [16]. ETS is illustrated by the inhibition of various defense pathways including those induced by SA, JA or by stabilization of ubiquitin ligases dependent protein degradation that prevent HR. Plant response against pathogen attack differs according to the lifestyle of the pathogen. Biotrophic pathogens generally induce the accumulation of ROS and an induction of SA signaling pathway activating the MAP kinase cascade to produce HR and SAR [8,9,17]. Necrotrophic pathogens, rather induce, ET and JA signaling pathways and accumulation of secondary metabolites, notably indole compounds derived camalexin and glucosinolates [8,9,18,19].

Many diseases are caused by seed transmission of pathogens, and seed immune responses have been poorly documented [20]. However, some studies reported that pathogen colonization to the seed could occur as if the pathogen became asymptomatic and would interact with the seed in a non-host relationship [21,22,23]. With regard to the importance of the seed germination into the reproductive success and the fitness of the species, it is hypothesized that the immune response of the seed could result from specific adaptive mechanisms, which differ from the defense strategies usually described in the leaf tissue. Among the seed-borne pathogens, the necrotrophic fungus *Alternaria brassicicola* (*A. brassicicola*), known as causal agent of *Alternaria* black spot disease in *Brassica* crop species [24,25,26], has been used to develop a patho-system with the *Arabidopsis* plant. This model patho-system opens perspectives to study the mechanism of pathogen seed transmission [24]. Previous molecular genetic approaches using rosette leaves allowed fine description of *Arabidopsis* defense mechanisms against *A. brassicicola* infection. Moreover, the insensitivity to fungal infection of the transgenic plants showing a reduced content in SA (*Nahg*) or a reduction of SA signaling (*sid2*) indicated that SA did not significantly contribute to resistance in leaf [27,28]. Meanwhile, transcriptomic approaches using the JA insensitive and the camalexin deficient mutants *coi1* and *pad3.1*, respectively, revealed the importance of JA signaling in the response to *A. brassicicola* [9,27,29]. During the plant-pathogen interaction, JA and ET signaling pathways synergistically induced activation of several defense genes, such as the plant defensin PDF1.2 [9,29]. Van Wees et al. [27] also underlined that camalexin could act as a direct antifungal toxin rather than a signaling compound in the defense response, although, extended studies using the camalexin defective mutants *pad1-5* and *pad4* indicated that camalexin was also able to activate some defense genes in response to *A. brassicicola* [27,30,31]. More broadly, the tryptophan-derived pathway including indole glucosinolates and their derived metabolites have also been related to defense mechanisms against necrotrophic fungal pathogen [9,31,32,33]. Their breakdown products, such as isothiocyanate, induce oxidative stress and are known to combat pathogen infection. However, necrotrophic fungi appear to take advantage of this oxidative stress, inducing cell death to successfully infect the host plant [9,33,34]. ROS generated by *Arabidopsis* peroxidases PRX33 and PRX34 increase the development of necrosis in plant tissues and contribute to fungal colonization success [35]. During plant-pathogen interaction, the virulence of the necrotrophic pathogen acts through different mechanisms [36] that modulate gene regulation. In this way, it is able to repress genes related to growth and development [18,19,37] and to hijack defense responses to their advantage [9,28,38,39]. The main question about what is happening in the germinating seed remains to be documented.

In this study, we used the *Arabidopsis/A. brassicicola* patho-system to describe at the RNA level the response of germinating seeds and seedlings to *A. brassicicola* infection. Healthy and infected seeds were compared at 3 days after sowing (DAS), corresponding to the pre-germinative stage, and 6 and 10 DAS, corresponding to post-germinating stages of seedling establishment and autotrophy set up. A set of 14 mutants, chosen for their deficiency in the identified *A. brassicicola*-responsive pathways based on the transcriptomic study, were characterized at early seedling stage for their germination behavior and their susceptibility to *A. brassicicola*. This work illustrates that the *Arabidopsis* germinating seed interacts with the necrotrophic pathogen by an unusual immune strategy, which differs from those characterized in the post-germinative stages of the plant’s life cycle.

## 2. Results

**Investigating *Arabidopsis* seed response in the interaction with the necrotrophic fungal *A. brassicicola*.** We sowed inoculated seeds at different reproducible inoculation concentrations to select the maximum rate of infected seeds, meanwhile maintaining the germinative quality of the seed lots. A range of inoculum concentrations between 10^2^ and 10^5^ conidia/mL was tested. The inoculum of 10^4^ conidia/mL exhibited maximal rate of seed infection without major impact on seed germination. Therefore, we selected this inoculum concentration for the following transcriptomic analysis [40]. The same inoculum concentration of 10^4^ conidia /mL was used in mutant analyses to characterize susceptibility phenotypes following fungal colonization.

**Germinating seeds (3 DAS) responded differently to *A. brassicicola* than 6- and 10-day old seedlings.** Using RNA-seq data, according to the designed experimental plan described by Ortega et al. [40], differentially expressed genes (DEGs) between the transcriptomes of healthy and *A. brassicicola* infected seeds were compared at 3 days, 6 days and 10 days after sowing (DAS), which corresponds, respectively, to germinating seeds, seedling emergency and establishment stages.

**Figure 1:**
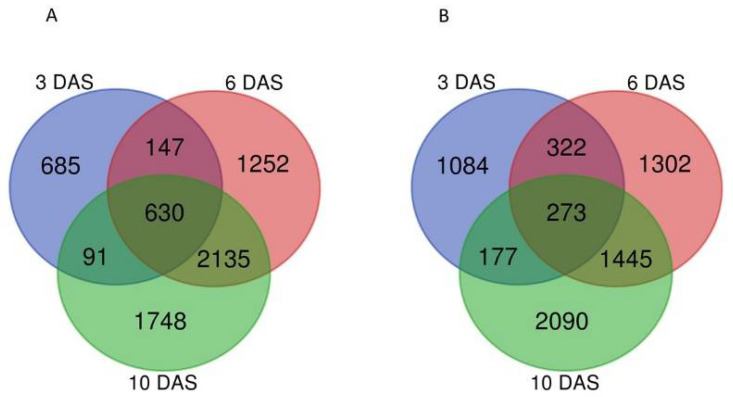
Comparison of DEGs induced by *A. brassicicola* at 3, 6 and 10 DAS. (**A**) Upregulated genes and (**B**) down regulated genes. The Venn diagrams show common and specific DEGs (log2FC  >  1 or  <  −1 and Benjamini-Hochberg adjusted *p*-value <  0.05) from healthy and *A. brassicicola* infected samples from pre-germinative stage (3 days after sowing, DAS), seedling establishment (6 DAS) and seedling autotrophy establishment (10 DAS).

Transcriptomic changes induced by the biotic interaction are indicated as Log2-ratio for gene expression of *A. brassicicola*-infected versus healthy plant in Supplementary Table S1. A cut-off of adjusted *p*-value (p-adj) < 0.05 highlighted 3409, 7506 and 8589 DEGs, respectively, at 3, 6 and 10 DAS (Figure 1A,B). Venn diagram comparisons of DEGs at 3, 6 and 10 DAS (Figure 1A,B) exhibited 273 and 630 genes, respectively, induced and repressed by *A. brassicicola* commonly at the three developmental stages. For up-regulated genes (Figure 1A), 81% and 84% of the identified DEGs in 6 and 10-day old seedlings differed from those identified at the germinating stage (3 DAS). For the two seedling stages (6 and 10 DAS), only 34% of the 6 DAS DEGs differed from those identified at 10 DAS. Regarding down-regulated genes (Figure 1B), 82% and 88% of the identified DEGs in 6 DAS and 10 DAS seedlings, respectively, differed from those from the germinating stage. When the two seedling stages were compared, only 48% of 6 DAS identified DEGs differed from those identified at 6 and 10 DAS. The higher rate of specific DEGs at the stage of germinating seeds strongly suggested that, upon germination, a large part of the *A. brassicicola*-related seed response can be distinguished from those identified at later seedling stages.

**Figure 2:**
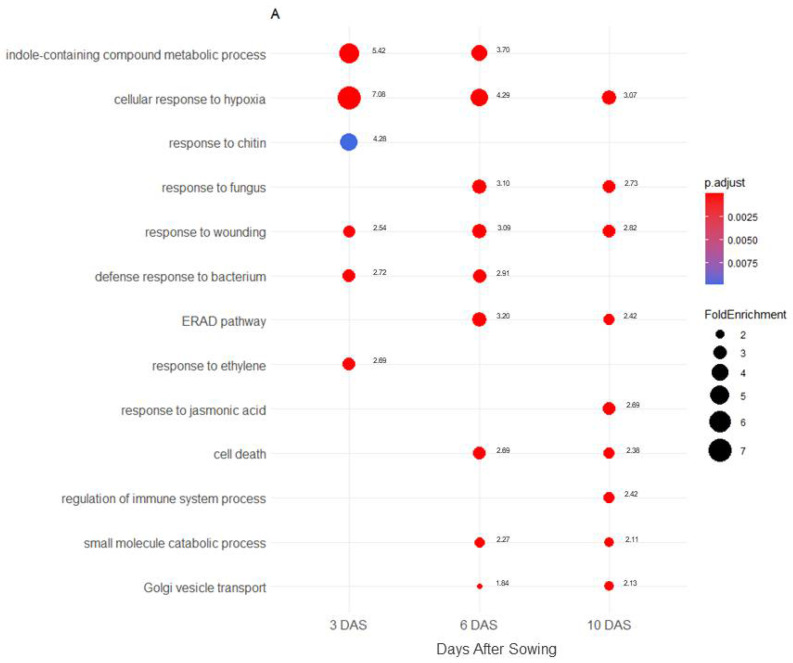

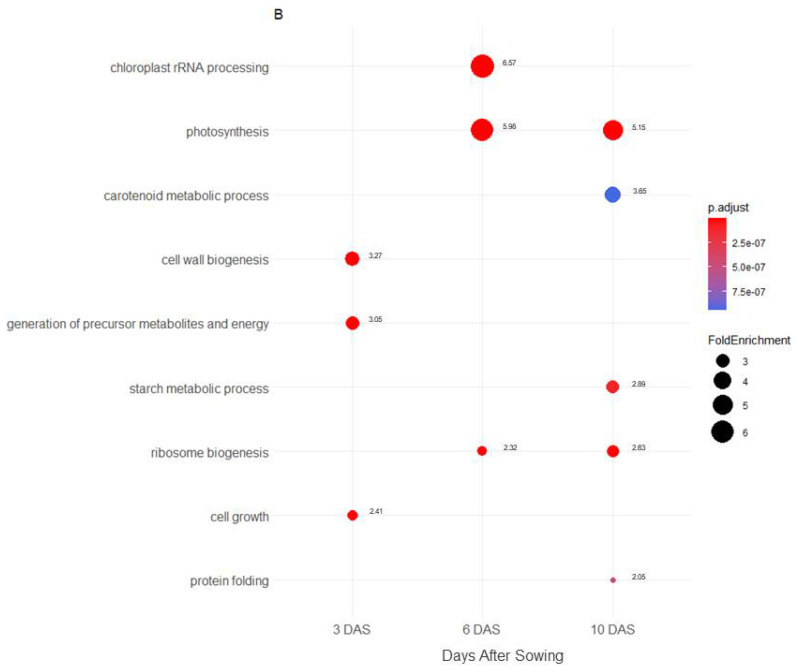
GO terms enrichment of DEGs induced by *A. brassicicola* in germinating seed and seedling. Analysis of enriched GO terms for biological processes of DEGs from the comparison of healthy and *A. brassicicola* infected conditions at 3, 6 and 10 DAS. (**A**) Top 13 functional enriched GO terms from upregulated genes; (**B**) Top 9 functional enriched GO terms from downregulated genes. Dot size represents fold enrichment indicating the ratio of input annotated genes frequency over the frequency of all annotated genes in that term. Color scale corresponds to adjusted *p*-values, obtained using the ClusterProfiler algorithm with a hypergeometric test and a Benjamini-Hochberg test [42]. Red color means higher significant enrichment than blue color.

**GO comparison of DEGs between healthy and *A. brassicicola* infected seeds at 3, 6 and 10 DAS**. To understand the response to *A. brassicicola* on plant gene expression in germinating seeds and at early post-germinating stages of the seedling, we performed Gene Ontology (GO) enrichment analysis. The other enrichment parameters such as gene ratio, enrichment factor and the total number of genes belonging to the respective GO class are indicated in Supplementary Table S2. The higher enrichments of assigned biological processes found based on the identified DEGs at 3, 6 and 10 DAS were illustrated for up and down-regulated genes (Figure 2A,B).

As shown in Figure 2A, among up-regulated genes in germinating seeds (3 DAS), those involved in response to hypoxia (fold enrichment index of 7.08), ethylene (2.69), metabolism of indole-secondary compounds (5.42), to chitin (4.28), to wounding (2.54) and to bacterium (2.72) were significantly enriched. In post-germinating stages at 6 and 10 DAS, response to hypoxia exhibited a fold enrichment index that decreased to 4.29 and 3.07, respectively, and the metabolism of indole secondary compounds decreased to 3.70 index enrichment at 6 DAS and was below the significant threshold index at 10 DAS. In the same way, the GO enrichment corresponding to the response to bacteria was maintained at 6 days (index of 2.91) and decreased below the threshold at 10 days. Both 6 and 10-day old seedlings showed similar enrichments, which differed from germinating seeds by an overrepresentation of up-regulated genes involved in response to fungus (enrichment indexes of 3.10 and 2.73, respectively), cell death (enrichment indexes of 2.69 and 2.38, respectively), small molecule catabolism process (enrichment index of 2.27 and 2.11, respectively), response to wounding (enrichment index of 3.09 and 2.82, respectively) and endoplasmic reticulum-associated degradation pathway (ERAD) (enrichment index of 3.20 and 2.42, respectively). Regarding the response to jasmonate and Golgi vesicle transport, respective enrichment indexes of 2.69 and 2.13 were found only in the 10-day-old seedlings. Interestingly, at the earliest stage corresponding to germinating seeds (3 DAS), the jasmonic acid (JA) pathway and regulation of the immune system GO terms were not over-represented in the gene set differentially induced by *A. brassicicola*.

Although these GO terms were described as the main response in the interactions with necrotrophic fungi [41], they displayed an over-representation only from 10-day old seedlings. This result allowed us to hypothesize that the interaction of the seeds with *A. brassicicola* during early germination differed from the canonical models described for the later developmental stages of the plant.

Regarding down-regulated genes by *A. brassicicola* (Figure 2B), GO enrichment analysis revealed that growing and energy metabolism were repressed by the necrotrophic interaction. Similar to the up-regulated genes, the repressed pathways identified in the germinating seed (3 DAS) clearly differed from those at the later seedling stages (6 and 10 DAS). Infected germinating seeds were characterized by down-regulation of generation of precursor metabolites and energy metabolism (enrichment index of 3.05) and cell wall biogenesis (index of 3.27), that included gene function involved in cell elongation and growth processes but also in abscisic acid (ABA) and JA metabolisms. At 6 DAS, repressed genes were related to chloroplast RNA processing GO term (enrichment index of 6.57). In 6- and 10-day old seedlings, down-regulation targeted photosynthesis activity (respective enrichment indexes of 5.98 and 5.15), and ribosome biogenesis (respective enrichment indexes of 2.32 and 2.63). Enrichment for *Alternaria*-related down-regulated genes belonging to carotenoid metabolism, starch metabolism, and protein folding GO terms were specifically represented (respective enrichment indexes of 3.65, 2.89 and 2.05) in the 10-day old seedlings. At 10 DAS, results suggested that *A. brassicicola* was able to repress the developmental program of the seedling through storage relocation and changes in transcriptional and post-transcriptional gene regulation.

Therefore, the GO analysis of the DEGs provide evidence that *A. brassicicola* seed infection caused increased expression of genes described to contribute to defense pathways, and on the other hand reduced the expression of genes involved in metabolic activity, growth and photosynthesis. Beyond the noted absence of inducing the JA pathway in germinating seeds, the identified responding pathways (Figure 2A), such as the induction of response to wounding and to catabolic process in seedlings and the repression of starch metabolism and cell wall biogenesis (Figure 2B) suggested that *A. brassicicola* in part would hijack the seed and seedling response to facilitate its colonization [43].

***A. brassicicola* widely induced salicylic acid (SA) response at early stage of the plant life cycle.** From the GO enrichment analysis, the fact that functional classes identified in germinating seeds (3DAS) were little or not represented in 6 and 10-days old seedlings may be due to the higher number of DEGs in post-germinative stages, which could dilute the contribution of some important pathways. For example, although the enrichment factor was below the selected threshold at 6 and 10 DAS for ethylene pathway, 118 and 105 genes were found to be induced, respectively (Supplementary Table S3). To obtain a more detailed view of the well-known defense pathways, we compared the number of their respective assigned up-regulated genes by *A. brassicicola* at each developmental stage. We extracted from the RNA-seq dataset all the assigned genes related to JA metabolic process (GO:0009694), indole-containing compound metabolic process (GO:0042430), glucosinolate (GSL) biosynthetic process (GO:0019761), phytoalexin biosynthetic process (GO:0052315), response to ROS (GO:0000302), response to ET (GO:0009723) and response to SA (GO:0009751). We identified 0 to 26 induced genes assigned to JA metabolism, 57 to 115 assigned to indole-containing compound, 0 to 62 assigned to response to ROS, 41 to 105 assigned to ET pathway and 92 to 269 assigned to SA response (Supplementary Figure S1 and Supplementary Table S3).

**Figure 3:**
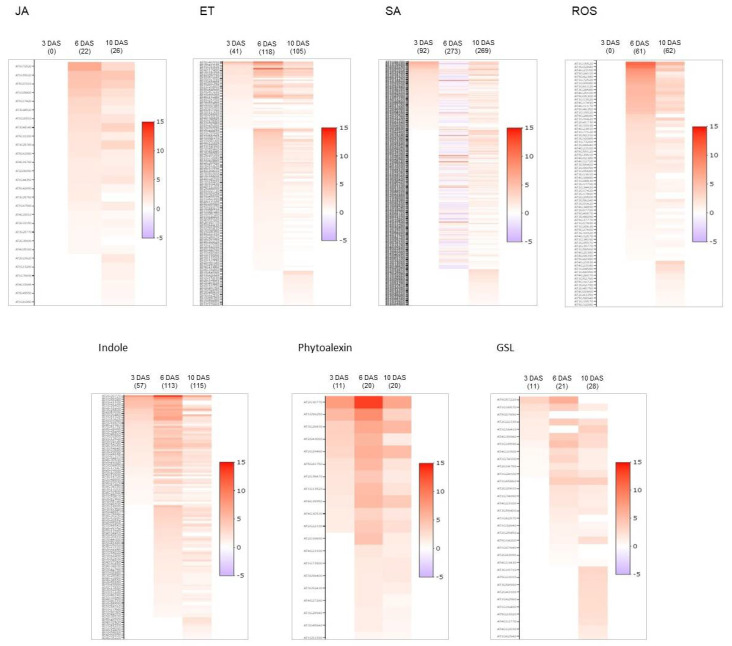
Heatmap of DEGs assigned to the main pathways and metabolism known to contribute to defense. Heatmap shows DEGs involved SA, JA, ET, indole and ROS pathways at 3, 6 and 10 DAS. Color scale is based on log2(FC): red for induced, white for non-differentially expressed and blue for repressed genes. The number of identified DEGS are indicated in brackets for each stage after sowing.

Heatmap of DEGs assigned to the SA, JA, ET, indole and ROS pathways (Figure 3) highlighted the importance of these pathways during *A. brassicicola* infection in germinating seeds and in post-germinating stages of the seedling. All the heatmap profiles exhibited a clear switch in the regulated gene networks between germinating seeds and seedlings. Very few genes of these pathways were found to be induced by the presence of *A. brassicicola* in germinating seeds (3 DAS). For instance, we observed that any genes from ROS or JA pathways were induced before the latter stages of seedling establishment at 6 and 10 DAS. Besides, the 6 and 10-day-old seedlings exhibited an activation of many genes related to ROS, ET and SA. Interestingly, the JA pathway was significantly induced only from 10 DAS and the number of induced genes remained negligible with 26 induced genes compared to the 269 related to the SA pathway.

**Unexpected *A. brassicicola*-related susceptibility phenotypes in defense pathway mutants**. It is noteworthy that gene annotation of most of the identified up-regulated genes described biological processes that were potentially contributing to plant resistance mechanisms. To investigate the physiological significance of the main defense pathways identified by the transcriptomic analysis, we performed phenotyping of available *Arabidopsis* defective mutants in GSL content in the seed, such as the *qk0* mutant (deficient for GSL biosynthesis) [44] and the *gtr1gtr2* (deficient for GSL transport to the seed) [45], in camalexin such as *pad3* [46], in the JA response such as *coi1* [47] and *jar1* [48] and in SA pathway such as *Nahg* and *npr1* [49]. We also included in the analysis deficient mutants in autophagy component such as *atg8* [50], a gene potentially involved in program cell death that was found induced by *A. brassicicola* in our RNA-seq dataset. In addition, some mutants altered in mucilage composition and structure such as *cesa5* [51], *mum2* [52], *csla2* [53] and *gl2* [54] were included. These latter mutant lines have impairments in genes that were shown to be repressed by *A. brassicicola* and have a structural and/or biochemical contribution to the interface contact between the seed tissue and the pathogen, making them good candidate genes for the entry of necrotrophic fungus into plant/seed tissues.

**Figure 4:**
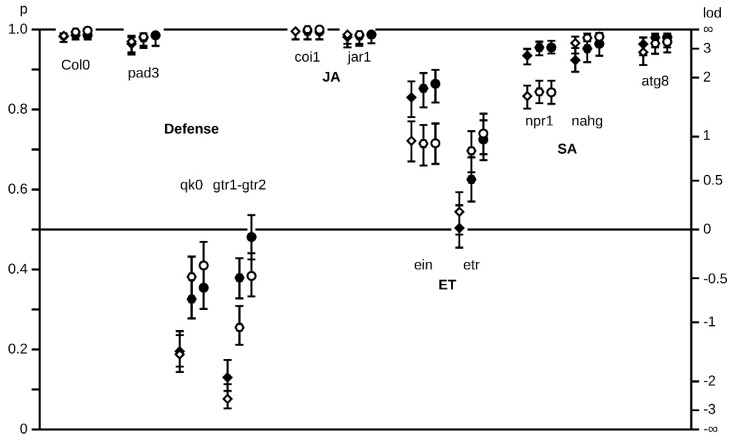
Seed germination rates of mutant phenotypes. Seed germination of defense defective mutants in healthy (non- infected seeds, withe dots) and infected (*Alternaria* infected seeds, black dots) conditions. Germination rates at 3, 6 and 10 DAS in *Arabidopsis* wild-type (Col-0) seeds and in the defective mutants (from a Col0 background) *pad3-1 (pad3), qk0 (qk0), gtr1gtr2 (gtr1gtr2), coi1-2 (coi1), jar1 (jar1), ein2 (ein), etr1 (etr), Nahg (nahg)* and *atg8 (atg8)*. Germination percentages are shown on the left scale and log odd ratios on the right scale. Error bars correspond to (non-symmetrical) 95% confidence intervals.

Germination rates of healthy and *A. brassicicola*-infected seed lots were first deter-mined at 3, 6 and 10 DAS in the mutants impaired in defense pathways (Figure 4). As previously described for the WT Col-0 [40], *A. brassicicola* inoculum at 10^4^ conidia per mL did not affect seed germination of Col-0 (Figure 4). Compared to the wild-type seeds, germinations of most of the mutants were not or only slightly altered in their maximal germination rate (Gmax), which was reached between 3 and 6 DAS. However, we noticed that germination rate was dramatically reduced for the *qk0* and the *gtr1gtr2* mutants, which did not contain any GSL in the dry mature seed, as well as for *ein2* and *etr1* mutants, which were impaired in the ethylene pathway. After 10 DAS, the maximum germination rate reached nearly 40% for the GSL-deficient mutants and 70% for mutants impaired in the ethylene pathway. It should be noted that, except in the case of the *ein2* mutant, the germination of these mutants was delayed as illustrated by their Gmax, which was reached only at 10 DAS. The contribution of ethylene in stimulating seed germination is well described [55,56]. Nevertheless, the low rates of seed germination in the GSL deficient mutants have never been reported. We also observed that storing GSL deficient mutant seeds at room temperature increased their germination capacity (not shown). This result suggested that the GSL defective mutants displayed a dormant phenotype. It is also noted that *A. brassicicola*-infected seed lots of *ein2* and *gtr1gtr2* mutants displayed significant higher germination rates compared to healthy seeds.

**Figure 5:**
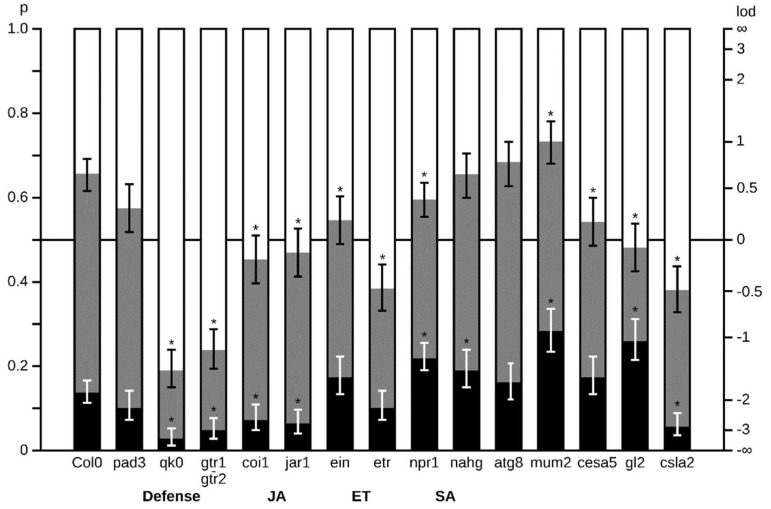
Rates of seedling colonization by *A. brassicicola* and of necrosis disease in defense and mucilage defective mutants. Seedling infestation at 10 DAS in *Arabidopsis* wild-type (Col-0) seeds and in the defective mutants (Col-0 background) *pad3-1 (pad3), qk0 (qk0), gtr1gtr2 (gtr1gtr2), coi1-2 (coi1), jar1 (jar1), ein2 (ein), etr1 (etr), Nahg (nahg), atg8 (atg8), mum2-1 (mum2), cesa5 (cesa5), gl2-1 (gl2), csla2.1 (csla)*. White color: No *A. brassicicola* mycelium present. Grey color: mycelium is present but there is no necrosis. Black color: both *A. brassicicola* mycelium and *A. brassicicola* caused necrosis are present. Probabilities are shown on the left scale and log odd ratios on the right scale. Error bars correspond to (non-symmetrical) 95% confidence intervals. For necrosis probabilities, asterisks indicate probabilities that are significantly (*p* < 0.05) different compared to Col-0 wild-type.

To validate our results, we evaluated the *A. brassicicola* seedling colonization and the necrosis symptoms in the different mutant seeds that germinated. Seed colonization and symptoms of the mutants were quantified at 3, 6 and 10 DAS from inoculated seed lots (Supplementary Table S4). A representative view of resistance/susceptibility to *A. brassicicola* shown for 6 DAS (Figure 5) highlighted six phenotypic profiles. The *pad3* and *atg8* mutants exhibited similar colonization and necrosis rates compared to the WT control and did not exhibit a particular susceptibility or resistance phenotype. The *mum2* deficient mutant impaired in mucilage expansion upon seed hydration was slightly but significantly more susceptible in term of *A. brassicicola* infection and necrose rates. The mutant *gl2* involved in mucilage pectin biosynthesis exhibited a lower rate of seedling infection and higher rate of seedling necroses. The *cesa5* mutant, impaired in the organization of adherent mucilage, exhibited a reduced rate of *A. brassicicola* colonization. The *csla2* defective mutant to adherent mucilage exhibited resistant phenotype with lower rates of *A. brassicicola* infection and necroses. Likewise, the *jar1* and *coi1* insensitive mutants to methyl-jasmonate, as well of the *qk0* and *gtr1gtr2* GSL deficient mutants exhibited a similar resistance phenotype to *A. brassicicola* with low rates of seedling infection and necroses. In the *ein2* and *etr1,* ET deficient mutants, the necrose rate was not significantly different; nevertheless significant higher resistance to colonization by *A. brassicicola* was observed. On the other hand, the *Nahg* and *npr1* deficient mutants in SA pathway displayed a significant higher susceptibility to *A. brassicicola* in term of necrosis.

From this mutant analysis, there is evidence that repression of genes involved in mucilage metabolism by *A. brassicicola* interfered with the strategy for fungal transmission through germination. Interestingly, among all mutants in the different defense pathways, only SA defective mutants exhibited higher susceptibility to *A. brassicicola*, which is surprising considering that SA is generally not described as the responding defense pathway triggered by necrotrophic attack [27,28]. The resistance phenotypes of JA, ET and GSL deficient mutants as well as the lack of susceptibility of the camalexin deficient mutant are also inconsistent with the described mechanism of plant defense against necrotrophic pathogens [9,27,29].

## 3. Discussion

**Figure 6:**
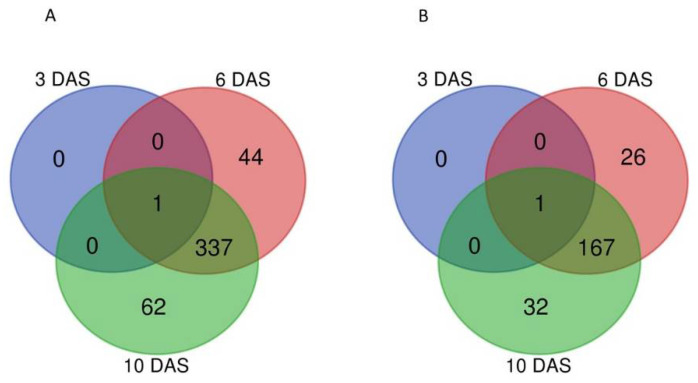
Comparison of DEGs induced by *A. brassicicola* identified by van Wees et al. [27]. Number of genes induced by *A. brassicicola* in rosette leave [27], were compared with upregulated genes in germinating seeds and seedlings (at 3, 6 and 10 DAS respectively). The number of common DEGs to those identified by van Wees et al. [27] are shown in the Venn diagram. (**A**) Comparison with genes induced by *A. brassicicola*; (**B**) Comparison with genes induced by *A. brassicicola* and related to the JA pathway.

Because the seed quality is determinant for reproductive success, it is hypothesized that specific adaptive mechanisms could control seed immune response. Despite that seed germination is the strategic phase of the life cycle in controlling the transmission of pathogens to the crop, plant-pathogen interaction has been poorly described at this specific developmental stage. Here, we illustrate the *Arabidopsis* seed response to the necrotrophic pathogenic fungus *A. brassicicola* by using a without a priori RNA-seq approach. The number of differentially expressed genes between healthy and infected tissues was shown to be high in germinating seed (3409 at 3 DAS), and increasing at the two post-germinating stages of seedling establishment (7506 and 8589 at 6 and 10 DAS respectively). Moreover, as shown in Figure 1, the rate of overlapping gene regulation between germinating stage (3 DAS) and the post-germinative stages (6 and 10 DAS) were lower compared to the two post-germinative stages. The GO enrichment assigned to DEGs induced by *A. brassicicola* seed inoculation (Figure 2) provided a global view of gene response to *A. brassicicola* in germinating seed that dramatically differed in 6 and 10 DAS seedlings, which strongly suggested that there was a switch in the *Alternaria*-related interaction mechanisms at early post-germinative phase. This switch in defense response between 3 and 6-day-old seedlings could be explained by the seed-to-seedling transition phase approximately occurring between 3 to 5 days after imbibition [57,58], switching the chromatin state program from embryonic- to vegetative-like tissues. We hypothesized that defense responses at 3 days after germination could reflect seed defense mechanisms more specific to the embryogenic phase. Instead, onwards after 6 days after germination, observed defense responses were more similar to those already described in vegetative parts. We highlighted that GO enrichment assigned to induction of JA pathway became significant only from 10 DAS (Figure 2A), with no JA-related gene expression in germinating seeds at 3 DAS (Figure 3). Previous mutant analyses and transcriptomic studies of *Arabidopsis* response to *A. brassicicola* using the rosette leave as model [27] displayed the importance of JA signaling in *A. brassicicola* resistance and *A. brassicicola* mediated gene induction: among the 505 identified *A. brassicicola*-induced genes, 264 were repressed in *coi1* mutant, thus illustrating the importance of the JA pathway in the rosette leaf immune response. Comparison with our data exhibited 1382 and 400 common up-regulated genes in germinating seeds and in 6 and 10 days-old seedlings (Supplementary Table S5 and Figure 6A), of which 226 were related to the JA pathway (Supplementary Table S5 and Figure 6B). The very low similarity of gene regulation found in seeds at 3 DAS compared to rosette leaves (i.e., one common gene) provides strong evidence that, upon germination, seed response to *A. brassicicola* differed dramatically from the mechanisms observed in later stage of the plant development. On the other hand, JA signaling seems to contribute to the response to *A. brassicicola* starting from post-germinative stage. Our hypothesis that the JA pathway does not contribute to the seed defense response and resistance was confirmed by mutant analyses. Contrariwise to the higher susceptibility phenotype of the *coi1* in *A. brassicicola*-infected rosette leaves [27], the *coi1* and *jar1* mutant exhibited a total resistance to *A. brassicicola* infection at the very early post-germinative stage (Figure 5).

Interestingly at 3 DAS, a high enrichment index in the DEGs assigned to the cellular response to hypoxia was observed. The contribution of this pathway in plant pathogen interaction is increasingly documented [59,60,61,62]. Hypoxia or pathogen responses act through ERF-VII (ethylene response factor VII) and WRKYs (WRKY transcription factors) involved in oxygen sensing and signaling. Nearly three quarters of these genes described to be related to both hypoxia and pathogen response in *Arabidopsis* [61] was found up-regulated in our RNA-seq dataset and particularly in germinating seeds (Supplementary Table S1). It is reported that both fungal pathogen and host are able to experience hypoxia during pathogenesis [62,63]. The response to low oxygen involves ethylene pathway factors and leads to competition with the pathogen for oxygen. This competition that is described as an example of “growth–defense tradeoff” [64] could be used advantageously by the seed that is the plant organ containing the lower oxygen content, (i.e., 1% in teguments) [62,65]. Seed germination is adapted to occur in a hypoxic context and the induced response to hypoxia in infected germinating seed could be at the same time detrimental for the growth of the pathogenic agent. In our analysis, the 100 to 167 identified DEGs involved in low-oxygen response included many protective enzymes against oxidative stress such as peroxidases or glutathione S-transferases. This detoxifying arsenal could interfere with ROS-induced cell death, which was not represented in the induced genes of germinating infected seeds (Figure 2A and Figure 3) and not observed phenotypically since no necrosis was observed before germination (Supplementary Table S4). We also suspect that the down-regulation of photosynthesis by *A. brassicicola* (Figure 2B) observed in 3 and 10 days-old infected seedlings would maintain the low level of oxygen because it is well known that oxygen production is linked to the photosynthesis activity. This could corroborate the decreasing but still highly enriched GO term of response to hypoxia observed from seedlings (Figure 2B). In hypoxia, gaseous compounds such as O_2_, NO, ET, CO_2_ or H_2_S change in level and crosstalk of H_2_S and NO with ABA to control stomatal closure (an important gateway for microorganism) have been reported [66]. It is not possible to decide whether response to low oxygen triggered by *A. brassicicola* is beneficial for the germinating seed or a way for the pathogen to propagate and induce diseases, but we documented here that oxygen-sensing takes an important part in *Arabidopsis/A. brassicicola* interaction at this early stage of the plant life cycle.

At 6 and 10 DAS, it was surprising to identify in infected seedlings many induced genes belonging to the SA pathway (Supplementary Figure S1 and Figure 3). SA is usually described to be involved in defense against biotrophic pathogens [8,9,17] and act antagonistically to the JA and ET agonist hormones that are, rather, attributed to the immune response in the interaction with necrotrophic agents [8,9,18,19]. We have described that JA pathway was poorly represented among the *A. brassicicola*-induced genes (Supplementary Figure S1, and Figure 2 and Figure 3). Usually, infected plants prioritize one of the defense responses, either JA/ET or SA depending on the type of pathogen [67,68,69]. Nevertheless, there are some reports that pathogens could also induce a harmless defense response as a strategy to repress the antagonistic pathway that could have been harmful to it [70,71,72].

A hypothesis is that, at the early post-germinating stage, SA would mediate susceptible responses (SRs) to *A. brassicicola* as it has been described for the induction of JA/ET-mediated SRs during plant development [73,74]. Plant SR [42] is not a passive loss of resistance but is rather considered as an active host cooperation induced by the pathogen, which contributes to propagation and disease. We hypothesize that in response to *A. brassicicola*, the young *Arabidopsis* seedlings prioritized SA pathway to the detriment of the JA pathway known as the main defense response against necrotrophs. Metabolic quantification of the hormones JA and SA, of their precursors and down-products in infected germinating seeds will make it possible to verify our theory. In the pathogen-induced susceptible responses (SR), numerous physiological processes have been reported such as the control of stomatal aperture, plant cell wall modifications, program cell death, transport of water, photo-assimilates and primary metabolism and response to abiotic stress. All of these physiological responses have been found among *Alternaria*-responsive genes in our data set (Supplementary Table S1) or are parts of the identified enriched GO terms (Figure 2 and Figure 3). It would be speculative to go into more detail in the analysis of all the genes potentially involved in the SR. We suspect that this opportunistic relationship varies continuously to adapt the pathogen to the physiology of the host so that seedlings would deploy other defense mechanisms. Particularly, there is a dynamic during the seed to seedling transition that we illustrated through the transcriptome profiles at 3, 6 and 10 DAS (Figure 3).

Cell death, response to ROS and ERAD pathway were induced by *A. brassicicola* at the post-germinative stage (Figure 2A). The absence of necrosis in infected seedling of GSL mutants strongly suggested that, in WT infected seedlings, the observed necrosis symptoms could be mediated by glucosinolate and glucosinolate-derived pathways that have been found to be induced by *A. brassicicola* (Figure 2 and Figure 3). It is thought that the necrotrophic pathogen induces PCD in host tissues to obtain nutrients [39] and cause disease. Our results also suggested that the GSL and GSL-derived pathway could be hijacked by *A. brassicicola* to spread in the seedlings and cause disease.

Repression of SR for disease and not for host colonization will induce plant tolerance without reducing fitness. This acquisition of tolerance would be a sustainable alternative strategy to the acquisition of resistance in plants [43]. Mutant analysis (Supplementary Table S4 and Figure 5) illustrated that necroses and fungal colonization could be disconnected. Moreover, the germinating seed that allow pathogen propagation to the seedling but does not exhibit any necrosis symptoms should also be a promising model for plant tolerance understanding.

## 4. Materials and Methods

### 4.1. Plant Material and Alternaria Strains

The *Arabidopsis* seeds used in this study are from Columbian (Col-0) genetic back-ground. Wild type genotype in Col-0 was obtained from the ABRC (Arabidopsis Biological Resource Center); the mutant lines *pad3-1* (Col-0; [46]), *gl2-1* (Col-0; [54]), *npr1* (Col-0; [49]), *jar1* (Col-0, [48]), *coi1-2* ( Col-0; [47]), *Nahg* ( Col-0; [75] ), and *atg8* ( Col-0; [50]) were obtained from the ABRC; the mutant lines *gtr1gtr2* (Col-0; [45]) and *qk0*, a quadruple mutant *cyp79B2 cyp79B3 myb28 myb29* (Col-0; [44]) were provided by Barbara Ann Halkier (DynaMo Center, Copenhagen, Denmark); the mutant lines *csla2-3* (Col-0; [53]), *cesa5* (Col-0; [51]), *mum2-1* (Col-0; [52]) were provided by Helen M. North (Institut Jean-Pierre Bourgin, Versailles, France); and the mutants *etr1* (Col-0; [56]) and *ein2* (Col-0; [56]) were provided by Françoise Corbineau (Laboratoire de Biologie du Développement, Paris, France, Paris France). Seeds were produced in controlled conditions using a culture chamber (Memmert ICP 750) at 20 °C with a 16-h photoperiod of light (120μmol photons m^−2^ s^−1^) at a relative humidity of 70% (RH). After seed maturation, *Arabidopsis* plants were submitted to water deprivation for three weeks and fully mature seeds were harvested by shaking into large paper bags. Then seeds were stored at 7 °C, 40% RH in airtight tubes (Eppendorf 2 mL, Sigma).

The *A. brassicicola* strain Abra43 [76] was used in this study. For routine culture, Abra43 was grown and maintained on potato dextrose agar (PDA).

### 4.2. Surface Sterilization and Seed Infection

Seeds (batch of 12 mg) were surface-sterilized by incubation in 1 mL of 30% bleach (*v/v*) for 7 min, followed by 7 min incubation in 1 mL of 80% Ethanol (*v/v*), and washed five times with 1 mL of sterile deionized water. The seeds were then dried for 5 h on a blotting paper in a Microbiological Safety Cabinet (SafeFAST Premium, FAST-ER). *Alternaria* strain was applied on sterilized seeds. The inoculum was prepared with *A. brassicicola* strain (Abra43) to obtain solutions with concentrations of 10^2^, 10^3^, 10^4^ and 10^5^ conidia.mL^−1^. For seed inoculation, 1 mL of the solution at the appropriate conidia concentration was added to 15 mg of seeds. After 1 h of mixing, the inoculated seeds were dried for 5 h on a blotting paper in a Microbiological Safety Cabinet. The non-inoculated seeds used as a control were subjected to the same treatment, replacing the conidial solution with water.

### 4.3. Phenotyping

Seed germination analyses were performed in microplates using the ScreenSeed automate according to the conditions described by Merieux et al. [77]. Incubation was performed inside a thermo-regulated incubator (Memmert ICP 750) regulated at 22 °C (±1 °C). Four replicates were measured in each condition analyzed and a minimum of 100 seeds per repeat was analyzed. Scoring was determined at 3, 6 and 10 days after incubation. Evaluating of the *Alternaria* propagation and of necrosis symptoms were carried out by manual scoring of samples sown in Petri dishes (Greiner Bio-one, diameter 9 cm) using a binocular magnifier (Olympus SZX10). At 3, 6 and 10 days after incubation in a controlled growth chamber with day/night temperatures of 22 °C and a 16 h photoperiod, the disease started to extend and was evaluated according to a symptom index corresponding to the disease severity (seed Statistical analyses below). To minimize observation bias, the notation of each condition was validated when the same score was determined by three different observers.

The data were subjected to statistical analyses. For each condition, seeds and seed-lings were scored according to their growth and infestation stage. Data were converted into three sets of binomial data: germination (0 for non-germinated seeds, 1 for germinated seeds), fungal colonization (0 for seeds/plantlets devoid of fungus, 1 for seed plantlets with fungal mycelium) and fungal necrosis (0 when absent, 1 when present). Each set of binomial data were fitted in logistic models. 95% confidence intervals was then calculated for each log odds ratio. Log odds ratios were then converted into probabilities. The confidence interval was not calculated when obtained probabilities were 0 or 1. All data were statistically analyzed with RStudio version 4.2.0 using the following libraries: readxl, plyr and ggplot2.

### 4.4. Growth Conditions for RNA Extraction

RNA extraction was carried out from WT samples, either from healthy or inoculated with *A. brassicicola* seeds and sown in petri dishes containing 0.8% agarose (SIGMA) for 3, 6 and 10 days of incubation in a controlled growth chamber under a 16-h photoperiod (170 µmol photons m^−2^.s^−1^) at 22 °C (light period)/20 °C (dark period) and a constant 70% RH (standard conditions). 20 mg of seeds were used for each sample with 3 replicates per condition. RNA extraction was performed using NucleoSpin® RNA Plus kit (Macherey-Nagel). The RNA extraction protocol was adapted using 500 µL of Lysis Buffer LBP, 5 min for the first centrifugation, 150 µL of Binding Solution, 500 µL of LBP, an extra washing step using WB2 with a 5 min centrifugation. RNA quantity was measured with a NanoDrop ND-100 (NanoDrop Technologies) and quality (RIN > 6.5) was determined with a 2100 Bioanalyzer (Agilent Technologies). RNA was sent to Beijing Genomics Institute (BGI, https://www.bgi.com (accessed on 30 July 2021)), Hong Kong for cDNA library preparation and paired-end sequencing (PE100, 40 M) were performed using the DNBseq™ technology.

### 4.5. RNA Sequencing Bioinformatic Analyses

After quality control [78,79], plant high-quality reads were mapped on reference genome/transcriptome of *Arabidopsis* Araport 11 [80,81] using quasi-mapping alignment of SALMON, version 0.14.1 [82]. Differentially expressed genes (DEGs) between healthy and infected seeds were determined using DESeq2 algorithms [83]. Genes with log2FC  >  1 or  < −1 and Benjamini-Hochberg adjusted *p*-values < 0.05 were considered as differentially expressed.

Gene Ontology Analysis using GO enrichment terms was performed with Cluster-profiler 4.0 package [42] in RStudio version 4.2.0. To detect significantly enriched GO terms related to different biological processes, an adjusted *p*-value cut-off of <0.05 after Benjamini-Hochberg multiple testing procedure was applied. Gene annotation and GO terms were assigned according to the Araport11 annotation version of the *Arabidopsis* genome [80,81]. DEGs and GO enrichment results were illustrated in Venn Diagrams (http://bioinformatics.psb.ugent.be/webtools/Venn/ (accessed on 14 March 2022)) and heat-map using the ComplexHeatmap [84] and heatmaply packages [85] in R studio version 4.2.0.

## 5. Conclusions

We have compared the *Arabidopsis* responses to *A. brassicicola* infection at early stages of the plant life cycle to highlight similarities and differences with defense mechanisms usually illustrated from the rosette leaf model [27]. We provided a view of regulated pathways at the RNA level in germinating seeds and at the following early steps of seedling establishment and autotrophy. Mutant phenotyping was helpful in appreciating the contribution of the main defense pathways identified from the transcriptomic analysis. This allowed us to propose a hypothetical model for seed immune response during this strategic phase of plant development. We showed that the interaction at this starting phase of the plant life is different from those described at later developmental stages, potentially due to the seed-to-seedling transition phase. Particularly, the observed SA-JA imbalance at the expense of JA as well as the activation of response to hypoxia suggested a non-canonical competition in the interaction with the necrotrophic agent. Together, the induced response to hypoxia (Figure 2) and the dormancy phenotype (Figure 4) of GSL *A. brassicicola*-resistant mutants (Figure 4 and Figure 5) points to the existence of a tradeoff between germination/growth and defense mechanisms [20].

## Data Availability

The RNA-seq datasets is publicly available in the repository NCBI GEO, number GSE199977: https://www.ncbi.nlm.nih.gov/geo/query/acc.cgi?acc=GSE199977 (accessed on 15 May 2022) [40].

## Supplementary Materials

The following supporting information can be downloaded at: https://www.mdpi.com/article/10.3390/plants11131708/s1, Figure S1: Number of differentially expressed genes (DEGs) assigned to SA, JA, ET, indole and ROS pathways; Table S1: Differentially expressed genes (DEGs) between healthy and *A. brassicicola* infected samples at 3, 6 and 10 days after sowing (DAS); Table S2: Enriched GO terms analysis of differentially expressed genes (DEGs) between healthy and *A. brassicicola* infected conditions at 3, 6 and 10 days after sowing (DAS) and their respective statistics; Table S3: Heatmap for the number of differentially expressed genes (DEGs) assigned to SA, JA, ET, indole and ROS pathways at 3, 6 and 10 days after sowing (DAS); Table S4: Quantification of seed colonization and necrosis symptoms in mutants at 3, 6 and 10 days after sowing (DAS); Table S5: Comparison of differentially expressed genes (DEGs) induced by *A. brassicicola* identified in van Wees et al. [27].
