# Supplementary material for: Seed Transmission of Pathogens: Non-Canonical Immune Response in Arabidopsis Germinating Seeds Compared to Early Seedlings against the Necrotrophic Fungus Alternaria brassicicola
Source: Plants (Basel). 2022 Jun 28;11(13):1708. doi: 10.3390/plants11131708 (PMC9269218; doi:10.3390/plants11131708)
Supplement: Supplementary file 1 [file plants-11-01708-s001.zip › Supplementary Figure S1. Heatmap Number of DEGs _GO defense.pdf]

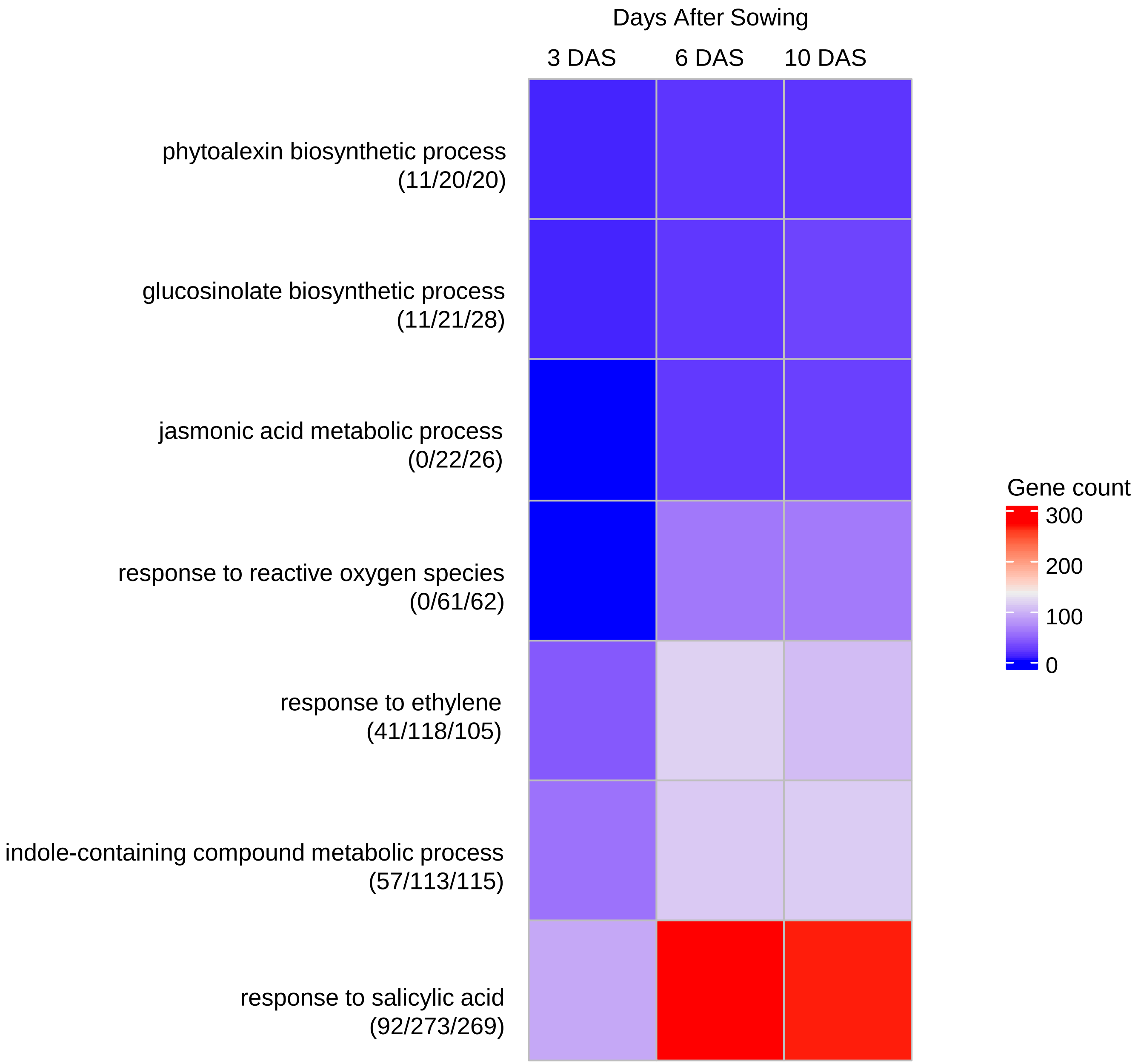

Supplementary Figure S1: Number of differentially expressed genes (DEGs) assigned to SA, JA, ET, indole and ROS pathways. Heatmap shows the number of DEGs assigned to these biological processes at 3, 6 and 10 days after sowing (DAS). Colour scale is based on the number of genes counted for each GO term, the scale colour goes from blue to red, starting at 0 and ending at 273 for our data.
